# Chondroitin Sulfate Impairs Neural Stem Cell Migration Through ROCK Activation

**DOI:** 10.1007/s12035-017-0565-8

**Published:** 2017-05-05

**Authors:** Layla T. Galindo, Mayara T. V. V. Mundim, Agnes S. Pinto, Gabrielly M. D. Chiarantin, Maíra E. S. Almeida, Marcelo L. Lamers, Alan R. Horwitz, Marinilce F. Santos, Marimelia Porcionatto

**Affiliations:** 1 Department of Biochemistry, Laboratory of Neurobiology, Universidade Federal de São Paulo, Rua Pedro de Toledo, 669 – 3o andar, São Paulo, SP 04039-032 Brazil; 2 Physiopathology Laboratory, Butantan Institute, São Paulo, 05503-900 Brazil; 3 Department of Morphological Sciences, Universidade Federal do Rio Grande do Sul, Porto Alegre, 90050-170 Brazil; 4 Department of Cell Biology, University of Virginia School of Medicine, Charlottesville, 22903 USA; 5 Department of Cell and Developmental Biology, Biomedical Sciences Institute, Universidade de São Paulo, São Paulo, 05508-000 Brazil

**Keywords:** Neural stem cell, Cell migration, Chondroitin sulfate, Traumatic brain injury, RhoA, Rock

## Abstract

Brain injuries such as trauma and stroke lead to glial scar formation by reactive astrocytes which produce and secret axonal outgrowth inhibitors. Chondroitin sulfate proteoglycans (CSPG) constitute a well-known class of extracellular matrix molecules produced at the glial scar and cause growth cone collapse. The CSPG glycosaminoglycan side chains composed of chondroitin sulfate (CS) are responsible for its inhibitory activity on neurite outgrowth and are dependent on RhoA activation. Here, we hypothesize that CSPG also impairs neural stem cell migration inhibiting their penetration into an injury site. We show that DCX+ neuroblasts do not penetrate a CSPG-rich injured area probably due to Nogo receptor activation and RhoA/ROCK signaling pathway as we demonstrate in vitro with neural stem cells cultured as neurospheres and pull-down for RhoA. Furthermore, CS-impaired cell migration in vitro induced the formation of large mature adhesions and altered cell protrusion dynamics. ROCK inhibition restored migration in vitro as well as decreased adhesion size.

**Electronic supplementary material:**

The online version of this article (doi:10.1007/s12035-017-0565-8) contains supplementary material, which is available to authorized users.

## Introduction

In the adult mammalian brain, neuroblasts from the subventricular zone (SVZ) travel through the rostral migratory stream towards the olfactory bulb, where they differentiate and integrate into the local circuitry [1–3]. Injury alters neurogenesis and stimulates neuroblast migration from the neurogenic niche to the injured area [4, 5]. Interaction of neural stem cells (NSC) with extracellular matrix (ECM) components is critical for migration, and external stimuli are transduced into cytoskeletal rearrangements that influence neuroblast migration by the action of RhoGTPases [6].

After a trauma to the brain, injured neurons present a limited capacity to regenerate due to the formation of a glial scar, which acts as a barrier for axons and neurite regrowth [7]. Glial scars are produced mainly by reactive astrocytes, oligodendrocytes, and microglia. These cells produce axonal growth inhibitory molecules, such as chondroitin sulfate proteoglycans (CSPG), Nogo, myelin-associated glycoprotein (MAG), and oligodendrocyte-myelin glycoprotein (OMGp) [8]. CSPG comprise a heterogeneous class of proteoglycans that includes, in the brain, RPTPβ, phosphacan, NG2, brevican, aggrecan, and neurocan. These membrane and ECM proteoglycans play important roles during CNS development, including control of axonal outgrowth and guidance [9, 10], directing neuronal precursor migration and regulating Purkinje cell differentiation and maturation in the developing cerebellum [11]. The CS side chains of CSPG are responsible for the inhibitory activity, and degradation of CS by the action of the bacterial enzyme chondroitinase ABC attenuates CSPG inhibitory activity and promotes axon regrowth [12–14]. Recent studies identified receptor protein tyrosine phosphatase sigma (RPTPσ) and Nogo receptor family members (NgR) as CSPG receptors that act through binding to CS [15–17].

Inhibition of neurite outgrowth mediated by CSPG depends on RhoA activation [16, 18, 19], and RhoA regulates maturation of cell-matrix adhesions and cell contractility through activation of myosin II [20]. Cell adhesion, contractility, and signaling help to polarize migrating cells and allow directional motility. Integrins, paxillin, and FAK (focal adhesion kinase), among other proteins, generate the signals that regulate directed cell migration [21].

In light of the importance of CSPG/CS in axonal growth inhibition and RhoA activation [8, 19, 22], we hypothesized that a similar mechanism may occur during neural stem cell adhesion and migration. Here, we report how CS regulates adult NSC migration in vitro, describing changes in protrusion formation and adhesion dynamics. Also, we suggest that these processes are mediated by RhoA/ROCK signaling.

## Material and Methods

### Animals

Adult male C57BL/6 mice used for all experiments were maintained under a 12 h light/dark cycle with access to water and food ad libitum. All experimental procedures and animal handling performed were approved by the Committee for Ethics in Research from Universidade Federal de São Paulo and University of Virginia Animal Care and Use Committee and followed international guidelines for care and use of experimental animals (http://www.iclas.org).

### TBI Model and Tissue Preparation

Adult 12-week-old male C57BL/6 mice were anesthetized with intraperitoneal injection of ketamine chloridrate (66 mg/kg) and xylazine (32 mg/kg) mixture (Dopalen, Brazil). Traumatic brain injury (TBI) was performed according to previously described protocol [23]. Briefly, a metal needle was chilled by immersion on isopentane on dry ice and was inserted four times into mice motor cortex (stereotaxic coordinates from bregma: AP +0.198 mm; ML +0.175 mm; DV −0.15 mm) [24]. Fourteen days later, mice were anesthetized and intracardially perfused with 4% paraformaldehyde (PFA) in 0.1 M PBS. Brain was removed from skull, postfixed in 4% PFA overnight at 4 °C, submersed in 30% sucrose at 4 °C, and frozen using liquid nitrogen. Cryostat coronal sections (20 μm) were collected on silanized slides (Superfrost slides, Fisher Scientific, USA) and prepared for immunofluorescence staining for detection of CSPG and DCX (marker for neuroblasts).

### Neurosphere Assays and Transfection

NSC were obtained from the SVZ of 6-week-old C57BL/6 mice and cultured as neurospheres as previously described [25]. Complete medium composition was DMEM:F12 1:1 (Gibco, USA), 2% B27 supplement (Gibco), 20 ng/ml EGF (Sigma, USA), 20 ng/ml FGF2 (R&D Systems, USA), 1% penicillin/streptomycin (Gibco), and 5 μg/ml heparin (Sigma). Neurospheres were ready to passage when the majority of them was about 100–150 μm in diameter [26]. Neurospheres were dissociated with trypsin and gently triturated to get single-cell suspension that was expanded as secondary spheres, used as single cells or used for nucleofection. Experiments were performed with cultures between passages 3 and 10.

For migration assay, glass coverslips were covered with 10 μg/ml poly-l-lysine (Sigma) for 30 min at room temperature, washed three times with 0.1 M PBS, dried on air, incubated with 50 μg/ml laminin (Sigma) for 30 min at 37 °C, washed with DMEM (Gibco), and finally incubated with 40 μg/ml CS-A sodium salt from bovine trachea (Sigma) for 4 h at 37 °C and washed with DMEM. All coverslips coated with CS were previously coated with laminin. Neurospheres were plated in complete medium, followed by incubation for 20 min at 37 °C in a CO_2_ incubator (to allow cell adhesion) prior to the treatment with 10 μM of ROCK inhibitor Y27632 (Santa Cruz, USA) or Nogo-66 (1–40) antagonist peptide (NEP1–40, Sigma). Migrated distance was defined as the extent of cell migration measured from the border of the neurosphere and the cell final position. Distance measurements were performed blind by an unbiased observer using ImageJ (http://rsbweb.nih.gov/ij/).

For total internal reflection fluorescence (TIRF) assay, neurospheres were dissociated and 3–5 × 10^6^ cells were nucleofected (Amaxa Biosystems, Germany) with 5 μg of paxillin-GFP plasmid [27], according to the manufacturer’s protocol (A-033) for adult neural stem cells. Immediately after nucleofection, cells were transferred to a 6-well culture plate with pre-warmed complete medium and incubated at 37 °C in a CO_2_ incubator for 24 h.

### Pull-Down

Active RhoA was measured using pull-down assay kit from Cytoskeleton (USA). Neurospheres were plated on laminin or laminin + CS precoated plates and 3 h after plating, neurospheres were washed with ice-cold PBS 0.1 M followed by harvesting with 1× Cell Lysis Buffer supplemented with 1× Protease Inhibitor Cocktail. NSC protein extract (240 μg) was incubated at 4 °C for 1 h with 50 μg of rhotekin-RBD beads, which binds specifically to GTP-Rho protein. Bead samples were resuspended in 20 μL Laemmli buffer. Total extract (15 μg), His-Rho control protein (20 ng), and bead samples were subjected to SDS-PAGE (12%) and transferred to a nitrocellulose membrane (GE Healthcare, UK). The membrane was blocked with 5% BSA (Sigma-Aldrich, USA) in TBS-T (TBS, 0,01% Tween® 20) at room temperature for 1 h and then incubated for 18 h at 4 °C with anti-RhoA monoclonal antibody (1:500, Cytoskeleton) or B-actin (1:10,000, Millipore, Germany). The nitrocellulose membrane was washed three times with TBS-T and subsequently hybridized with horseradish peroxidase-conjugated anti-mouse IgG (1:5000, Santa Cruz Biotechnology, EUA) at room temperature for 1 h. After three washes with TBS-T, immune complexes were visualized by adding Luminata Forte Western HRP Substrate (Millipore, Germany) and chemiluminescent signal was acquired on Odyssey FC® (LI-COR Biosciences, USA). Molecular size of immunoreactive bands was determined by Kaleidoscope prestained protein standards (Bio-Rad, USA).

### Microscopy

#### Migration Speed and Protrusion Dynamics

NSC as single cells were plated on laminin or laminin + CS glass-bottomed dishes in complete medium containing 25 mM Hepes, allowed to adhere for 20 min at 37 °C in a CO_2_ incubator and imaged in an inverted phase contrast microscope (TE-300; Nikon, Japan) equipped with 37 °C heater and controlled by Metamorph software (Molecular Devices, USA). For migration speed and directionality, time-lapse images were obtained with a ×10 objective at 5-min intervals for 18 h and each cell was individually tracked using ImageJ software. For protrusion dynamics, time-lapse images were acquired at 5 s during 30 min, and for each protrusion, a line (5 pixels wide) was drawn along regions oriented in the protrusion direction and perpendicular to the lamellipodial edge. Protrusion parameters were quantified by kymograph [28], using ImageJ software. The results were plotted in a graph where the *Y*-axis is the distance reached by the lamellipodium along that line, and the *X*-axis is time.

#### Adhesion Dynamics

NSC expressing paxillin-GFP were plated on laminin or laminin + CS. After 40 min, images were acquired on a Total Internal Reflectance Fluorescent (TIRF) microscope (Olympus IX70, 1.45 NA oil Olympus PlanApo 660 TIRFM objective) fitted with a Ludl modular automation controller (LudlEletronic Products, USA) and controlled by Metamorph. Paxillin-GFP was excited with a 488-nm laser line of an Argon laser (MellesGriot, USA), and images were acquired at 3-s intervals for 8 min and analyzed using ImageJ software. The leading edge was zoomed in (150%) and the size of paxillin-positive adhesions within focal adhesion sites was measured when matured and before disassembly.

### Immunohistochemistry

Fixed coronal brain sections, neurospheres, or single NSC were permeabilized with 0.1% Triton X-100 for 10 min, blocked with 5% fetal bovine serum in 0.1% Triton X-100 for 1 h at room temperature, and then incubated with primary antibodies at 4 °C overnight. Incubation with the appropriate secondary antibodies or FITC-Phalloidin (1:200, Sigma) and DAPI (1:10,000, Molecular Probes, USA) was performed at room temperature for 1 h. Glass slides were mounted using Fluoromount G mounting medium (Electron Microscopy Sciences, USA). Brain images were captured on a Leica TCS SP8 confocal microscopy using LASAF software (Leica, Germany), and NSC images were captured on an Olympus FluoView 300 confocal system using the FluoView software (Olympus, Japan).

Primary antibodies: mouse anti-CSPG (1:250, Abcam, USA); guinea pig anti-DCX (1:1000, Millipore, USA); chicken anti-GFAP (1:500, Abcam). Secondary Antibodies (Invitrogen, USA): Alexa Fluor 594-conjugated goat anti-mouse IgG (1:300); Alexa Fluor 488-conjugated goat anti-guinea pig IgG (1:1000); Alexa Fluor 488-conjugated goat anti-chicken IgG (1:500).

### Statistical Analysis

Data are presented as mean *±* SEM and analyzed using the unpaired Student’s *t* test by the use of Prism v5.0 software (GraphPad Software, USA). Statistical significance was set at *p* < 0.05.

## Results

### A CSPG-Rich Environment Impairs NSC Migration into the Injury Site

**Fig. 1:**
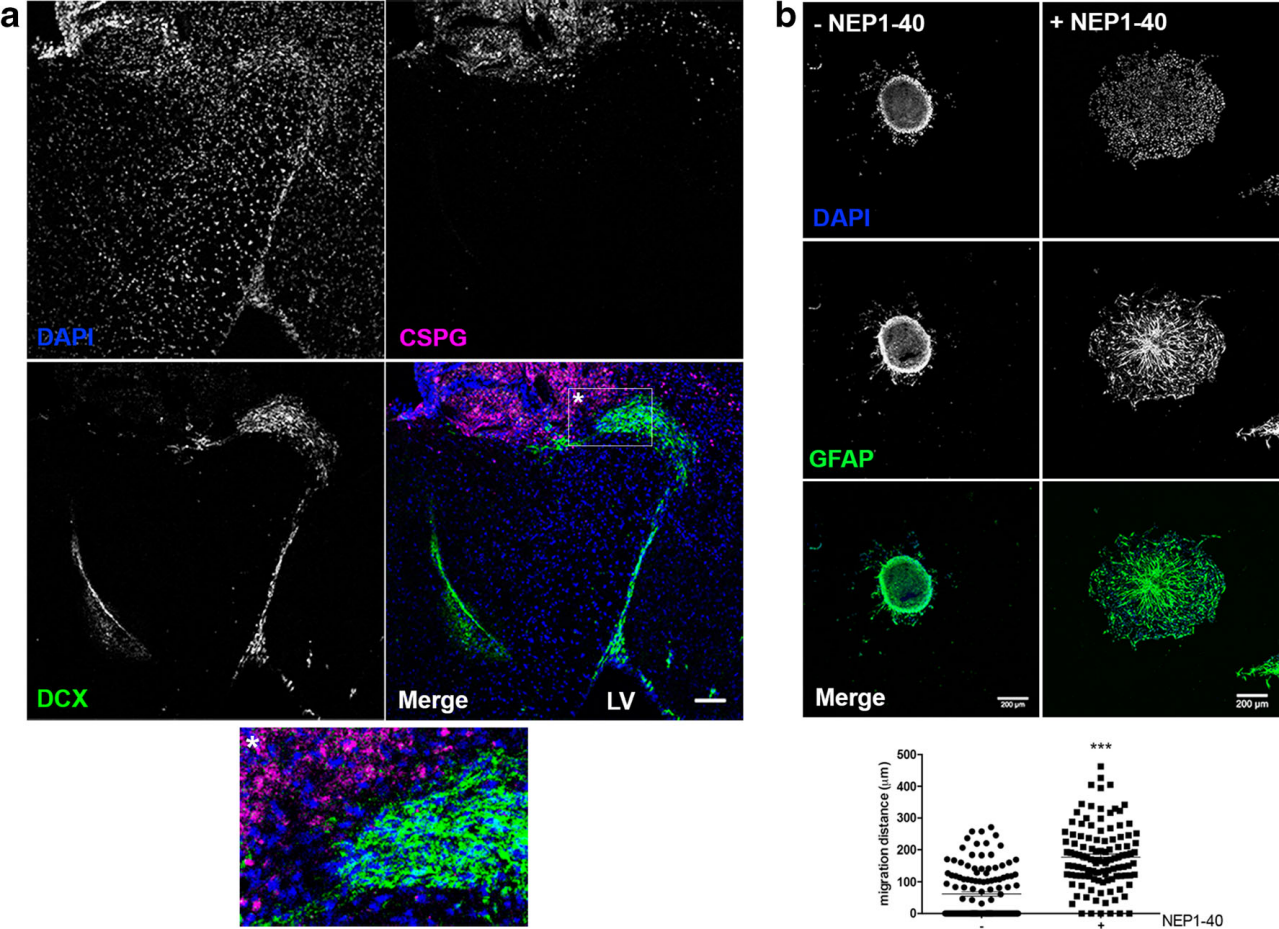
CSPG impairs NSC penetration into the injury site after TBI and acts through NgR. **a** Two-week postinjury glial scar was already formed and NSC migration was evaluated. DCX+ neuroblasts (*green*) leave their niche at the lateral ventricle (LV) and migrate towards the injury site characterized by high expression of CSPG (*magenta*). NSC are prevented to enter the proteoglycan-rich regions, accumulate at the injury border, and migrate around this area (detail). *Scale bar* at 100 μm. **b** NSC cultured as neurospheres were plated on laminin + CS with or without the NgR inhibitor NEP1–40. CS inhibits NSC migration and decreases the distance traveled by the cells. In the presence of NEP1–40, NSC migrated longer distances (*p* < 0.0001), suggesting that the inhibition of migration promoted by CS is mediated by NgR. Data were collected 18 h after neurospheres were plated. Number of neurospheres analyzed: with NEP1–40 = 115; without NEP1–40 = 123. NSC were immunolabeled with GFAP (*green*) and nuclei stained with DAPI. *Scale bar* at 200 μm (Color figure online)

Following injury, migratory DCX+ neuroblasts leave the SVZ niche located at the lateral ventricle wall and migrate towards the injury guided by chemokines, ECM components, and blood vessels [5, 29, 30]. To assess whether neuroblast migration into the CSPG-rich scar was impaired, we performed a TBI model to the adult murine motor cortex, and 2 weeks after TBI, DCX+ neuroblasts that migrated from the SVZ towards the injury were immunolocalized. DCX+ neuroblasts did not penetrate into the CSPG-rich injury site (Fig. 1), shown by immunostaining for CSPG core protein. As soon as the neuroblasts reach an area with high content of CSPG, cells pile up at the injury border and migrate around it.

To assess how CS impairs NSC migration in vitro, SVZ-derived NSC cultured as floating neurospheres were plated on laminin + CS, which induces an inhibitory substrate for neurite growth, and treated with NEP1–40, a Nogo-66(1–40) antagonist peptide which blocks signaling through Nogo receptor 1 (NgR1). NgR1 is implicated as a functional receptor for MAIs (myelin-associated inhibitors) [31, 32] and recently characterized as receptor for CSPG expressed by neurons [15]. NSC derived from neurospheres plated on laminin + CS and treated with 10 μM NEP1–40 migrated longer distances (average 180 μm) when compared to NSC plated on laminin + CS without NEP1–40 treatment (average 60 μm) (Fig. 1b). These data suggest NgR1 as a CS receptor which mediates impairment of NSC migration.

### CS Inhibits NSC Migration and Decreases Migration Speed In Vitro

**Fig. 2:**
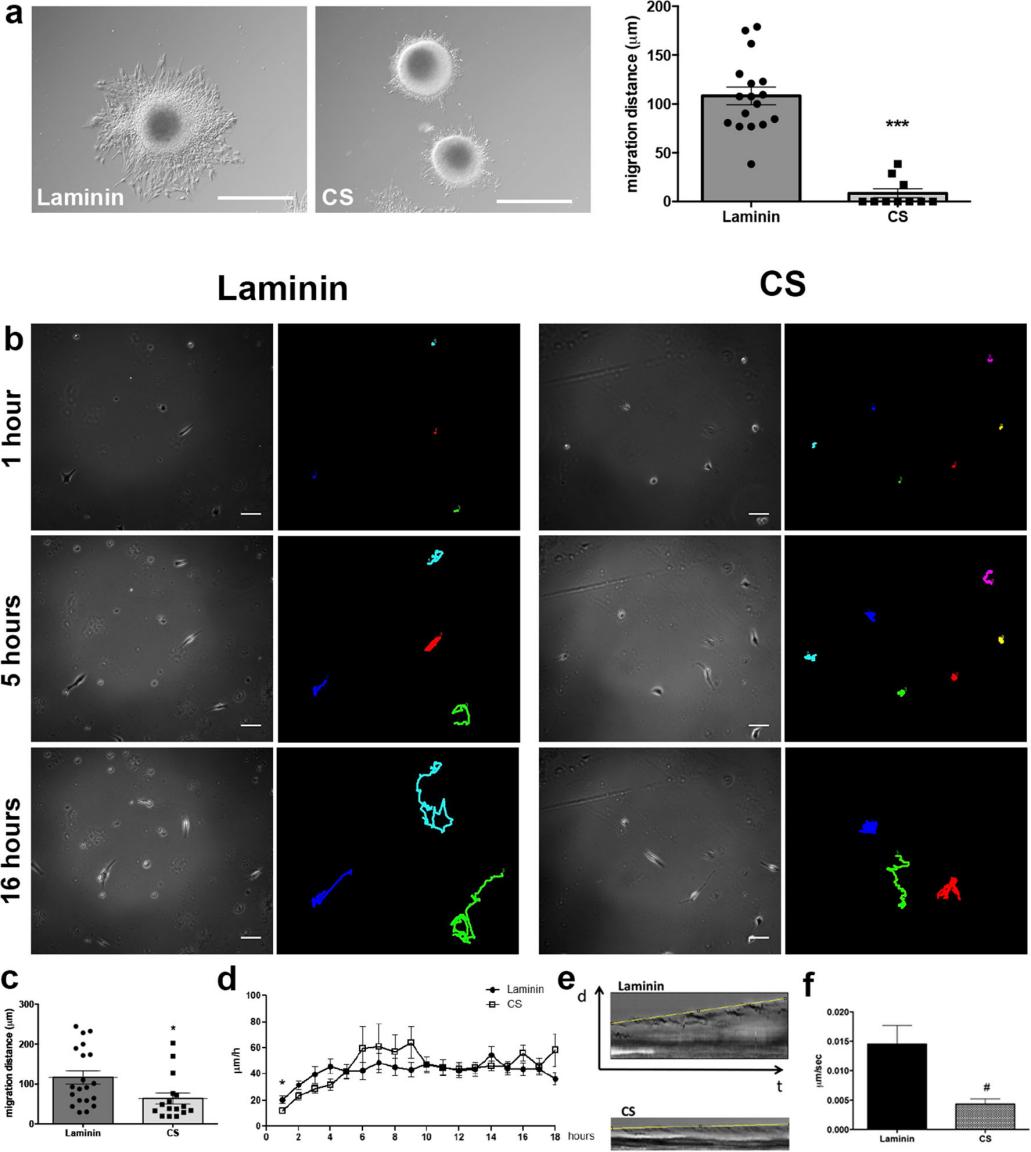
CS inhibits NSC migration, decreases speed, and alters protrusion, and adhesion dynamics in vitro NSC cultured as neurospheres were plated on laminin or laminin + CS. **a** CS impairs NSC migration and decreases the distance traveled by the cells (***p < 0.0001). Data were collected 18 h after neurospheres were plated. *Scale bar* at 200 μm. Number of neurospheres analyzed: laminin = 17; CS = 10. **b** NSC were plated as single cells, and images were captured every 5 min for 18 h. Cells are represented as *dots* and the migration routes as lines 1 and 5 and 16 h after start. *Scale bar* at 20 μm. **c** Quantification of migration from start to finish from NSC plated as single cells for 18 h represented on Fig. 2b (*p = 0.0230). Number of cells analyzed: laminin = 20; CS = 16. **d** NSC average speed during 18 h of migration. NSC are significantly slower within the first hour of migration (*p = 0.0181) and kept migrating lower over time although not reaching statistical significance. From the hours 6 to 9, cells migrated faster (not statistically significant) on laminin + CS than on laminin, and from hours 10–18, migration speed was similar in both substrates. Number of cells analyzed: laminin *n* = 20; CS *n* = 15. **e** Kymographs of NSC plated on laminin and laminin + CS. Protrusion progressions are outlined by the *yellow line*. NSC protrusions on laminin + CS are nonproductive with no retraction or progression. **f** NSC protrusions formation speed is slower on CS (#*p* = 0.0167). Number of protrusions analyzed: laminin = 22; CS = 17. At least three independent movies were acquired for each condition. *d* distance, *t* time. *Scale bar* at 200 μm (Color figure online)

In order to elucidate how CS might influence NSC migration and to evaluate NSC migratory behavior in response to CS, neurospheres were plated on laminin only, a permissive substrate, or on laminin + CS and measured migration distance and speed. When adhered to laminin, cells migrate out of the neurosphere, and in contrast, NSC migration was greatly inhibited when neurospheres were plated on laminin + CS in comparison to laminin alone (Fig. 2a; *p* < 0.0001). Furthermore, all cells migrating from neurospheres plated on laminin + CS migrated less than 10 μm, whereas the average migration distance of cells plated on laminin was 100 μm. These results show the inhibitory properties of CS on NSC migration, corroborating with the in vivo results presented in Fig. 1.

Based on the observation that CS inhibited NSC migration in vivo and in vitro, we wondered whether CS also affected the speed of migrating cells. Neurospheres were dissociated and NSC were plated as single cells on laminin + CS covered glass bottom plates. Images were acquired at 5-min intervals for 18 h. NSC migrating on laminin + CS migrated less distance than those on laminin (*p* = 0.0230) (Fig. 2b, c) and moved significantly slower in the first hour (average speed 11.8 μm/h; *p* = 0.0181) when compared to cells plated on laminin (average speed 20.3 μm/h). In the following hours, although not reaching statistical significance (hour 2 *p* = 0.083; hour 3 *p* = 0.109; hour 4 *p* = 0.058), the inhibitory effect of CS was consistent among experiments, as cells kept on moving slower on laminin + CS than on laminin (Fig. 2d and Online Resources 1 and 2), and there was an increase although not statistically significant, in migration speed of cells on laminin + CS up to 9 h. From the tenth hour on, migration speed was equivalent on laminin + CS and on laminin, suggesting that CS inhibits the initiation of migration in vitro, and inhibition is not sustained for longer periods of time.

### CS Alters NSC Protrusion and Adhesion Dynamics

Protrusion formation and adhesion dynamics are early migratory events. Following the observation that CS inhibits NSC migration and decreases migration speed in vitro, the question was whether CS would affect these key events in cell migration. The presence of CS in the substrate caused a 50% decrease in the number of cells displaying three or more protrusions when compared to cells grown on laminin alone (data not shown), and the protrusions were more stable with increased ruffling. Kymography revealed that protrusions of NSC plated on laminin + CS exhibited no progression, and the speed of protrusion formation was significantly slower (*p* = 0.0167) compared to protrusion formed by cells plated on laminin that induced persistent protrusion progression (Fig. 2e, f). These data suggest impairment on cell adhesion properties induced by CS.

**Fig. 3:**
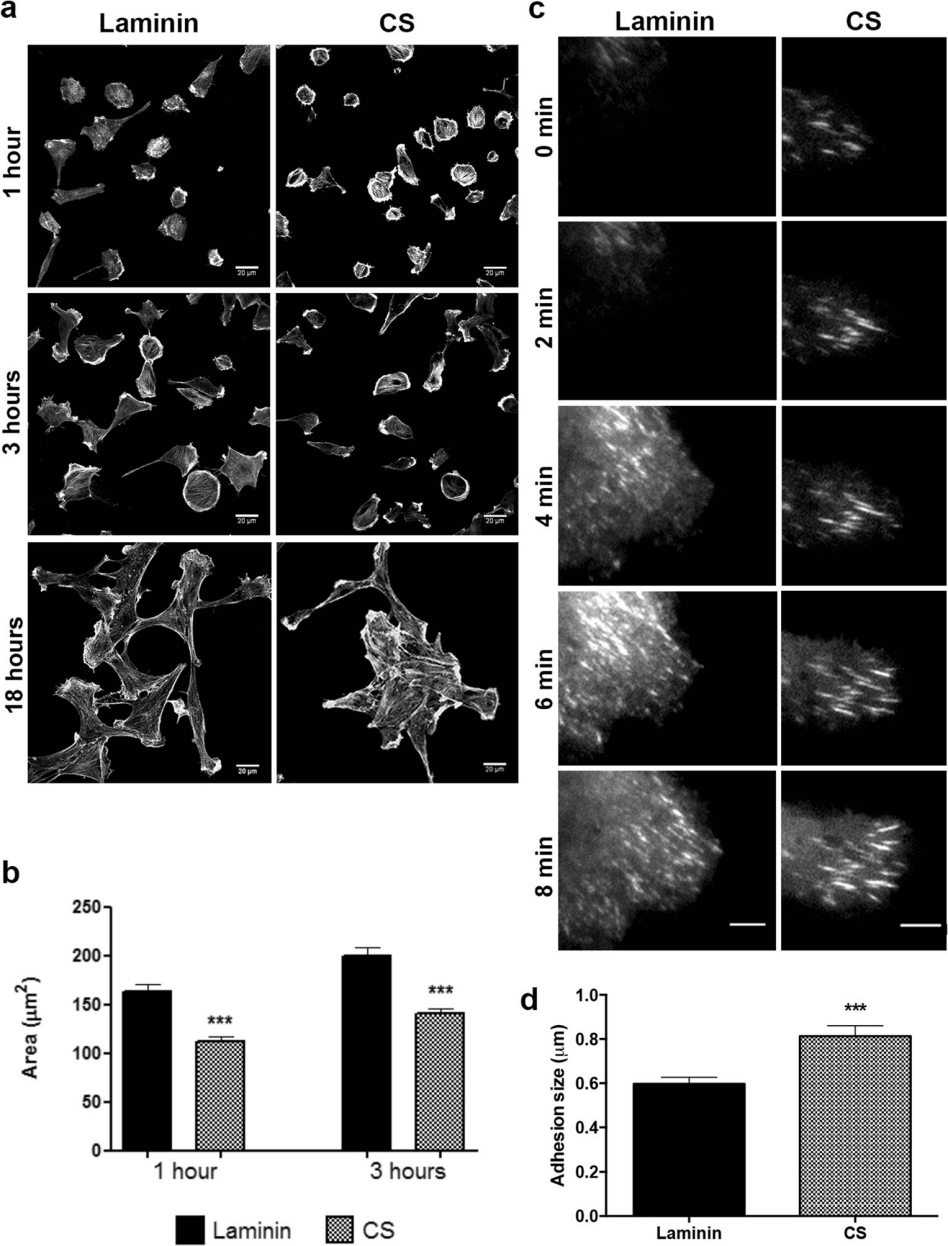
CS promotes formation of large and elongated adhesions. **a**, **b** NSC were plated on laminin or laminin + CS for 1 and 3 h, fixed, and stained with FITC-phalloidin. CS induced NSC to show a smaller spreading area in comparison to cells plated on laminin. At least 30 cells were measured for each condition in three independent experiments. After 18 h, NSC formed clusters on laminin + CS, whereas cells were isolated when plated on laminin only. *Scale bar* at 20 μm. **c**, **d** Cells were nucleofected with GFP-paxillin and plated on laminin or laminin + CS, and pictures were captured using TIRF microscope. CS induced formation of larger and elongated adhesions on NSC when compared to laminin (***p = 0.0001). *Scale bar* at 6 μm. Number of adhesions analyzed: laminin = 77; CS = 71

Next, NSC were plated on laminin or laminin + CS substrates for 1 and 3 h, followed by fixation, FITC-phalloidin staining, and measurement of cell area (Fig. 3a). At all time points, NSC plated on laminin + CS displayed smaller area in comparison to cells on laminin (Fig. 3b), and the majority of cells remained rounded showing many prominent stress fibers, which is usually associated with inhibited migration. After 18 h, individual NSC spread on laminin and formed clusters on laminin + CS, suggesting that the inhibitory environment induces NSC to migrate along or towards each other and not exhibit exploratory behavior (Fig. 3a). After 18 h, cells were overlapping and it was not possible to define boundaries in order to have an accurate measure.

To evaluate adhesion formation and dynamics, NSC expressing paxillin-GFP were imaged using TIRF microscopy 40 min after plating on laminin or laminin + CS. Forty percent of the adhesions produced by NSC plated on laminin matured into stable adhesions, and adhesions were productive with active turnover, whereas CS promoted the formation of large elongated and stable adhesions in approximately 57% of the adhesions near the cell leading edge, and adhesions presented no turnover, assemble and disassemble (Fig. 3c, d and Online Resources 3 and 4). All together, these data suggest that CS induces the production of stable protrusions and adhesions, which inhibits NSC spreading and migration.

### RhoA Mediates CS Inhibitory Effects on NSC Migration

**Fig. 4:**
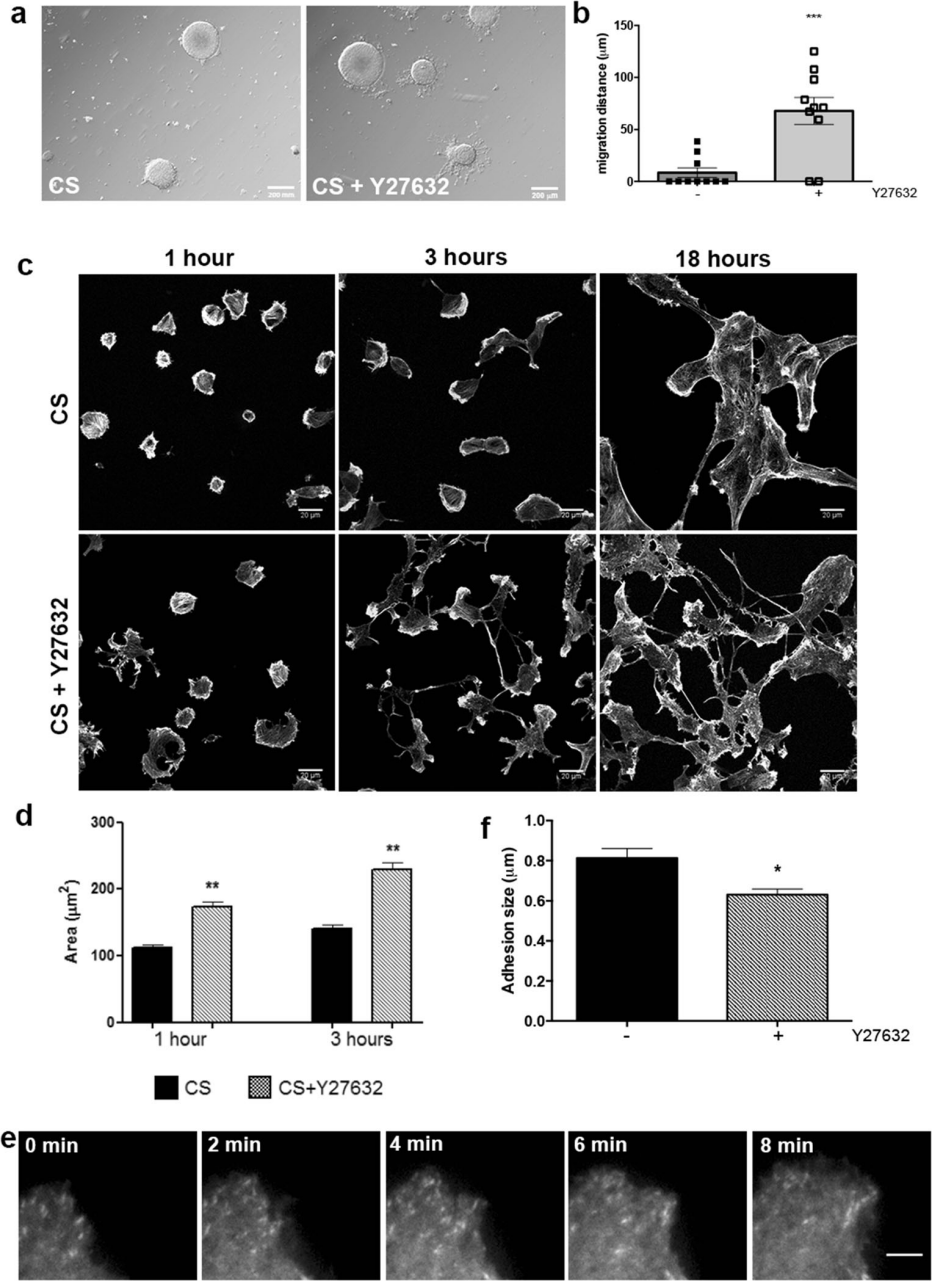
RhoA/ROCK inhibition promotes NSC migration on CS. **a**, **b** NSC migrate significantly longer distances in the presence of 10 μM of Y27632 on laminin + CS, 18 h after plating (***p = 0.0004). *Scale bar* at 200 μm. **c**, **d** Inhibition of ROCK increases NSC area 1 and 3 h after plating on laminin + CS (***p = 0.0001). At least 30 cells were measured for each condition in three independent experiments. Eighteen hours after plating, NSC do not form cell clusters on CS in the presence of Y27632, as it was observed without ROCK inhibition. **e**, **f** TIRF analyses revealed that adhesions were significantly smaller on cells plated on laminin + CS in the presence of ROCK inhibitor (**p = 0.0188). Number of adhesions analyzed: CS = 71; CS + Y27632 = 30. *Scale bar* at 6 μm

Signals from ECM and soluble factors regulate NSC migration, and most of these signals converge to RhoGTPases which regulate cytoskeleton reorganization and cell migration [6]. Treatment of neurospheres and NSC single cells with Y27632, an inhibitor of ROCK, reversed the inhibitory effects of CS on cell migration, suggesting that CS regulates RhoA/ROCK signaling pathway (Fig. 4a). Inhibition of ROCK leads to a significant increase in the distance NSC migrate out of the neurosphere (*p* = 0.0004) (Fig. 4b). Furthermore, NSC spreading area on laminin + CS substrate was larger in cells treated with ROCK inhibitor than in untreated cells (Fig. 4c, d). After 3 h of Y27632 treatment, some cells formed two or more protrusions and a long tail, and after 18 h, clusters of NSC plated on laminin + CS were not observed (Fig. 4c). Besides, while 70% of neurospheres plated on laminin + CS had no cells migrating, addition of Y27632 induced 100% of the neurospheres to have cells migrating more than 50 μm (data not shown). TIRF analyses also revealed that cells plated on laminin + CS in the presence of Y27632 produced significantly smaller adhesions than those produced by untreated cells (Fig. 4e, f and Online Resource 5).

**Fig. 5:**
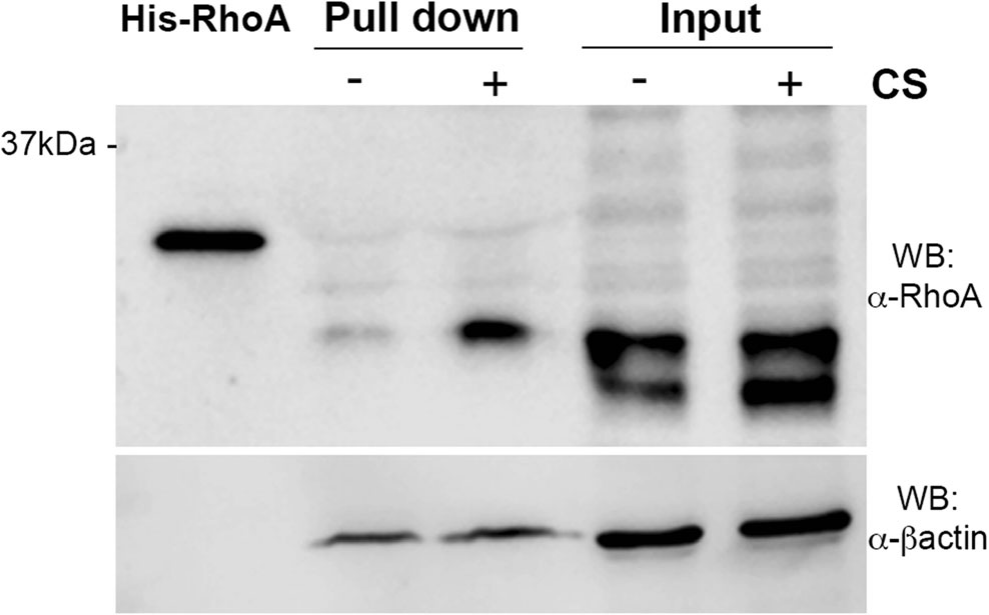
CS induces RhoA activation. Neurospheres were plated on laminin or laminin + CS for 3 h, and pull-down assay was performed to assess RhoA activity. Laminin + CS increased RhoA activation within 3 h after neurospheres were plated. One pull-down experiment was performed in triplicate

In order to determine if CS regulates RhoA/ROCK signaling pathway during NSC migration, we assessed the activation of RhoA/ROCK using pull-down assay to measure active RhoA (GTP-bound RhoA) 3 after neurospheres were plated on laminin or laminin + CS. RhoA activity was increased in neurosphere-derived cells plated on laminin + CS for 3 h (Fig. 5). These results provide additional insight that CS inhibits the initiation of NSC migration in vitro through RhoA/ROCK activation.

## Discussion

In the adult injured nervous system, components of the glial scar such as CSPG inhibit axon growth and regeneration [33]. The TBI-induced glial scar is a CS-rich environment that prevents SVZ-derived migratory neuroblasts from entering the injured area, reducing the chances for regeneration. Using in vitro experiments, we were able to show that CS acts through NgR and activates RhoA/ROCK signaling, decreases distance and speed of NSC migration and also induces the formation of large stable adhesions. Inhibition of ROCK allowed NSC migration and reversed CS effects on NSC, suggesting that CS inhibits NSC migration through RhoA/ROCK activation.

Cell proliferation and migration to the injury site are the initial steps in NSC recruitment to regenerate the injured tissue. Traumas to the CNS such as ischemia or TBI activate astrocytes and oligodendrocytes that, together with infiltrating blood cells, express cytokines and chemokines as part of the local inflammatory response. This localized increase in chemokines produces a gradient that attracts NSC from the neurogenic niche localized at the SVZ that migrate towards the injured area [5, 34, 35]. Expression of chemokines and their receptors is upregulated minutes after injury and can last days depending on the severity of the trauma [29].

We investigated neuroblast migration towards TBI 2 weeks postinjury and observed that there is extensive migration of those cells from the SVZ to the lesion site. However, the glial scar composed of OMGp, MAG, Nogo, and CSPG prevents neuroblasts from penetrating the injury [36, 37]. The glial scar is well characterized as a specific ECM composition that starts to form 7 days post-injury, is accompanied of massive astrogliosis, and prevents axons from entering the lesion as well as causing growth cone collapse due to filopodia retraction [38–40].

Recently, LAR (leukocyte common-antigen related phosphatase) and NgR have been described as CSPG receptors, both expressed by NSC. Dyck et al. [41] showed that knockdown of LAR and RPTPσ increases spinal cord NSC attachment, spreading, survival, differentiation, and proliferation on CSPG substrate in vitro. Furthermore, NgR1, NgR3, and RPTPσ knockout mice showed enhanced fiber regeneration following injury to the optic nerve [15, 19], NgR1 inhibition promoted NSC proliferation and differentiation [42, 43], and mild hypothermia combined with small interfering RNA silencing of NgR gene in NSC promoted NSC axonal outgrowth after SCI in rats [44]. Neurospheres plated on CS substrate in the presence of the NgR antagonizing peptide NEP1–40 were able to migrate, strongly suggesting that NSC inhibition of migration by CS is mediated by NgR followed by activation of RhoA/ROCK.

Several studies reported that the CS side chains are responsible for CSPG-mediated inhibition of neurite outgrowth and growth cone collapse, and treatment of neurons with chondroitinase ABC, an enzyme that degrades CS, abrogates the inhibition [12, 14, 45–47]; however, in vivo treatment with chondroitinase ABC increases significantly the inflammatory response in the injury site [12]. Based on those observations, we investigated the effects of CS on NSC migration in vitro*.*

Laminin is a glycoprotein that mediates cell adhesion and migration during development and wound healing, and is a permissive environment for NSC migration both in vivo and in vitro. NSC plated on laminin were able to migrate, whereas migration was impaired by the addition of CS. Considering that neurospheres behave as a 3-D environment for stem cells only when they are floating, when adhered to the plate cells migrate out of the sphere on a 2D substrate, similar to what happens to the dissociated cells. Although on the first hour, the speed of single cells migrating on CS (12 μm/h) is 60% of the speed of cells migrating on laminin (20 μm/h), the net traveled distance, measured as a straight line from start to finish, is inhibited by 55%, similar to what we observed for cells migrating away from the neurosphere. Moreover, NSC moved significantly slower during the first hour in the presence of CS, probably due to impairment of protrusion speed and stability, indicating that the inhibitory effect of CS is relevant for the initiation of the NSC migratory process. These results agree with Gu et al. [45] who demonstrated that treatment of embryo-derived neurospheres with chondroitinase ABC enhanced NSC migration in vitro.

Cell migration is a multistep process composed of leading edge protrusion, focal adhesion turnover, generation of traction forces, and tail retraction and detachment. NSC plated as single cells were unable to spread on CS surface, keeping a smaller area at all time points analyzed. Protrusion stability indicates that alterations in protrusion dynamics could be related to disturbances in adhesion formation. TIRF time-lapse images showed that CS induced the formation of large, stable adhesions. Assembly and disassembly of adhesions and cell detachment are essential for cell migration, and migration speed also relies on the strength of cell attachment [48–50]. Number and size of adhesions can influence cell migration. This is supported by the observation that fibroblasts lacking protein tyrosine phosphatase have an increased number and size of adhesions, culminating in a migration defect [51]. Our results suggest that as NSC adhesions on CS are larger than those formed on laminin and do not disassemble, cells have difficulty to detach from the CS substrate, hampering cell protrusion and migration.

Rac and Cdc42 are required at the leading edge, mediating the formation of lamellipodia and filopodia, respectively, whereas Rho and its effector ROCK are required for actin/myosin stress fibers assembly for adhesion maturation and detachment of the cell rear [21, 52]. The function of Rho/ROCK at the leading edge is less clear. However, our results suggest that CS induces the activation of RhoA in the initiation of cell migration in vitro, hampering migration process.

Here, we show that inhibition of ROCK significantly stimulated NSC migration on CS. Cells lost the rounded shape and after different periods of time assumed a migratory shape with a leading actin protruded edge, although some cells exhibited several protrusions and long tails, which persisted until the 18th hour. These morphological characteristics are consistent with previous studies, which reported a long cell tail in hematopoietic stem cells, leukocytes, and fibroblasts induced by treatment with ROCK inhibitor Y27632 [53–55]. Unlike what was described in these studies, Y27632 did not lead to impaired cell migration; on the contrary, it has decreased the adhesion sizes and stimulated NSC migration on the inhibitory substrate, partially reverting CS inhibitory effect. Similar results were observed with human keratinocytes that showed increased migration in the presence of Y27632 [56] and murine myofibroblasts (C2C12), cells that increased cell migration and speed and significantly reduced the size of adhesions when treated with ROCK inhibitor [57].

Inhibition of RhoA or ROCK leads to neural tissue regeneration after glial scar formation in spinal cord and optic nerve [18, 58]. ROCK inhibitor also enhanced NSC migration in vitro in SVZ explants cultured in Matrigel® [59]. Similar to other inhibitory molecules such as MAIs, CSPG axonal growth inhibitory effect depends on RhoA activation [19, 60]. Our results corroborate with that, as ROCK inhibition allowed NSC migration on CS.

In conclusion, repair to TBI or to other injuries to the CNS is challenging mainly due to glial scar formation, axonal growth inhibition, and cell survival. NSC-derived neuroblast migration to an injured site is an endeavor to repair. Here, we show that neuroblasts migrate towards the injury site but are prevented to enter into the lesion and glial scar rich in CS among other inhibitory molecules. Our in vitro experiments suggest that CS hampers NSC migration due to changes in cell protrusion and adhesion dynamics, which were recovered by inhibition of RhoA/ROCK or modulation of NgR activation.

## Electronic Supplementary Material

Online Resource 1 (AVI 3337 kb)

Online Resource 2 (AVI 5141 kb)

Online Resource 3 (AVI 3986 kb)

Online Resource 4 (AVI 311 kb)

Online Resource 5 (AVI 242 kb)
